# BCAM-AKT2 fusion protein promotes unfolded protein response driven oncogenic signaling in SKOV3 epithelial ovarian cancer cell line

**DOI:** 10.1371/journal.pone.0357882

**Published:** 2026-09-25

**Authors:** Hala Khazem, Ravid Solomon Zemler, Liron Vardi David, Dvir Reder, Shilhav Meisel Sharon, Shay Hantisteanu, Haim Werner, Ilan Bruchim

**Affiliations:** 1 Gynecologic Oncology Laboratory, Division of Obstetrics and Gynecology, Hillel Yaffe Medical Center, Hadera, Israel; 2 The Ruth and Bruce Rappaport Faculty of Medicine, Technion - Israel Institute of Technology, Haifa, Israel; 3 Division of Obstetrics and Gynecology, Hillel Yaffe Medical Center, Hadera, Israel; 4 Department of Human Molecular Genetics and Biochemistry, Gray Faculty of Medical and Health Sciences, Tel Aviv University, Tel Aviv, Israel; Mayo Clinic Rochester: Mayo Clinic Minnesota, UNITED STATES OF AMERICA

## Abstract

In view of the rapidly increasing evidence that gene fusion mutations are frequent drivers of cancer, a deeper understanding of the molecular and functional hallmarks of specific gene fusions in epithelial ovarian cancer (EOC) is essential. In this work we sought to identify the molecular signature of the BCAM-AKT2 fusion protein in ovarian cancer cell lines and to assess the biological impact of the novel fusion in these cells. Our *in-vitro* results show that the BCAM-AKT2 fusion protein is engaged in proliferative action within EOC cell lines. Furthermore, RNA-seq analysis shows that the effect of BCAM-AKT2 in SKOV3 cells is associated with activated unfolded protein response (UPR) and with the inhibition of interferon alpha and beta signaling pathways, pyropstosis and viral response in EOC cell lines. Overall, our results indicate that in SKOV3 cells, BCAM-AKT2 modulates the UPR signaling pathway, which is known to interact with oncogene and tumor suppressor networks during cancer development. These findings offer fresh insights suggesting that BCAM-AKT2 could serve as a promising target for therapeutic interventions in ovarian cancer.

## 1. Introduction

Epithelial ovarian cancer (EOC) is the most lethal gynecological malignancy in the world, accounting for 90% of all ovarian tumors [1]. Surgery and platinum-based combination chemotherapy treatments have increased the survival of patients over the past few decades; however, 75% of patients have a high rate of disease relapse and die within five years of diagnosis. In most EOC patients, the disease is diagnosed at advanced stages and has, therefore, a poor prognosis. Although most of the patients will respond to primary treatment, 80% will have recurrent disease and resistance to chemotherapy and biologic-targeted therapies [2]. EOC divides into two broad categories, designated type I and type II. Type I tumors are comprised of low-grade serous, low-grade endometrioid, clear cell and mucinous carcinomas. Type II tumors possess a more aggressive pattern of disease behavior. As tumors develop rapidly, they are usually widely dispersed at the time of presentation, resulting in a very poor prognosis for patients. Tumors of this type are comprised of high-grade serous, high-grade endometrioid, malignant mixed mesodermal tumors (carcinosarcomas) and undifferentiated carcinomas. Most of these cancer cells are genetically unstable (TP53 mutations predominate) and highly proliferative [3]. High-grade serous carcinoma (HGSC) is by far the dominant clinically diagnosed subtype among the type II neoplasms and accounts for 70–80% of deaths from all forms of ovarian cancer [4]. HGSC is characterized by genomic instability with a high rate of genomic rearrangements which make development of patient-specific targeted therapies even more difficult.

Chromosomal aberrations are a common event in tumorigenesis and they result, eventually, in the formation of fusion proteins. Fusion proteins usually contain functional domains derived from different genes that can affect transcription, DNA binding, or protein-protein interactions and thus are a key etiological factor in cancer [5]. Several fusion proteins were identified in recent years in different tumors, including ovarian cancer [6]. According to Mitelman and colleagues, it is estimated that 20% of cancer morbidity in the world is caused by translocations and gene fusions [7]. The oncogenic effects of fusion proteins are typically caused either by deregulating one of the involved proteins (e.g., by fusing a strong promoter to a proto-oncogene) or by generating proteins that have oncogenic functions (e.g., by constitutively activating a tyrosine kinase) [8]. Recent research has revealed that fusion genes possess intrinsic carcinogenic mechanisms, including damaging protein functional domains, causing mutagenesis, changing molecular subcellular localization, obtaining active or strong promoters, upregulating downstream effectors and more [9]. There is evidence that the disordered fragments in fusion proteins contribute to their carcinogenic properties whereas at the structural level breakpoints in fusion proteins tend to be in disordered regions [8]. From a clinical standpoint, it is generally believed that cancers associated with fusion proteins have a poor prognosis [10].

In 2015, Kannan et al identified a recurrent cancer-specific gene fusion in ovarian cancer. The rearrangement consisted of a fusion between basal cell-adhesion molecule (BCAM), a membrane adhesion molecule, and AKT2, a key kinase in the PI3K signaling pathway. This fusion was present in 7% of the 60 cancer patients tested, a significant frequency considering the highly heterogeneous nature of ovarian cancer. Kannan et al. provided evidence that BCAM-AKT2 is translated into an in-frame fusion protein in the patient's tumors. As a result, AKT2 fusion kinase is constitutively phosphorylated and activated as a functional kinase in cells. Unlike endogenous AKT2, whose activity is tightly regulated by external stimuli, BCAM-AKT2 escapes the regulation from external stimuli [11].

AKT has three isoforms (AKT1, AKT2, AKT3) that regulate many cellular processes, including metabolism, cell cycle, survival, protein synthesis and differentiation. The PI3K-PKB/Akt pathway is one of the most frequently mutated pathways in cancer [12]. There is an association between mutations of the PI3K pathway and tumor growth, metastatic spread and treatment resistance [13]. This study investigates the role of the BCAM-AKT2 fusion protein in driving the progression of ovarian cancer by examining its impact on cellular pathways within EOC cell lines.

## 2. Materials and methods

### 2.1. Cell lines and treatments

Human ovarian cancer cells lines OVCAR4 and KURAMOCHI were provided by Prof. Ruth Peretz (CRIR, Rambam health care campus). SKOV3 cell line was provided by Prof. Ilan Tsarfaty (Faculty of Medicine, Tel Aviv University). OVCAR4 cell line was maintained in DMEM: F12 HAM, KURAMOCHI cell line was maintained in RPMI medium, and SKOV3 was maintained in DMEM containing 2 mM glutamine and 100 μg/ml streptomycin. All cell lines were maintained in the presence of 5% CO2. All media were supplemented with 10% fetal bovine serum (FBS). All media and reagents were purchased from Biological Industries (Kibutz Bet-Haemek, Israel).

### 2.2. Transient transfections

OVCAR4, SKOV3 and KURAMOCHI cell lines were seeded in 6-well plates. Cells were transfected using X-tremeGENE HP DNA Transfection reagent (Roche, Merck) according to manufacturer’s recommendations. Briefly, one or two µg of DNA was added at a ratio of 1:2 to the transfection reagent diluted in 200 µl of OptiMEM™ (Gibco, Thermo Fisher Scientific). This mix was incubated for 15 minutes at room temperature and then titrated to cells for 48 hours. GFP empty vector was provided by Prof. Haim Werner (Gray Faculty of Medical and Health Sciences, Tel Aviv University) [14]. Flag-BCAM-AKT2 and Flag-AKT2 plasmids were provided by Laising Yen's lab (Baylor College of Medicine, Houston, Texas) [11]. The BCAM-AKT2 and AKT2 expression constructs used in this study correspond to FLAG-tagged plasmids (Flag-BCAM-AKT2 and Flag-AKT2, respectively). Therefore, the terms BCAM-AKT2 and Flag-BCAM-AKT2, as well as AKT2 and Flag-AKT2, are used interchangeably throughout the manuscript and refer to the same plasmid constructs used for transfection experiments.

### 2.3. XTT assay

Cell proliferation was monitored using an XTT cell proliferation assay kit (abcam cat# ab232856) following the protocols described by the manufacturer. XTT was conducted 3 days post-transfection of GFP (empty vector) or BCAM-AKT2 plasmid. Absorbance was measured with a spectrophotometer at a wavelength of 450 nanometers (SQ2 plate reader). Relative proliferation rate was calculated by normalizing the absorbance values of the experimental group to those of the GFP-transfected control group, which was set to 1 (100%).

### 2.4. Western blot analyses

Cells were grown on 6-well plates, washed twice with phosphate buffered saline (PBS) and incubated with lysis RIPA buffer (Tris 10mM, EDTA 1mM, NaCl 140mM, Triton X-100, SDS 0.1%, Na-Doc 0.1%, Protease Inhibitor) for 20 min on ice. The suspension was centrifuged at 13,000 rpm for 10 min, diluted with sample buffer and boiled for 5 min at 95C. Samples were electrophoresed through 10% SDS-PAGE at 200 volts, followed by electrophoretic transfer of the proteins to nitrocellulose membranes. After blocking with 5% non-fat milk, the blots were incubated overnight at 4C with antibodies against Flag (#F3165-Sigma-Aldrich 1:500 dilution), tubulin (#T6199-Sigma-Aldrich 1:1000 dilution), phospho-ERK 1/2 (dual phosphorylation of Thr 202 and Tyr 204, #sc-81492-Santa Cruz 1:500 dilution) or GAPDH (#MAB374-Sigma-Aldrich 1:1000 dilution). Then the blots were washed and incubated with a secondary antibody at a 1:10,000 dilution. Proteins were detected using the SuperMarker 2700 West Pico® Chemiluminescent Substrate (Pierce, Rockford, IL, USA). Each experiment was repeated three times and a representative experiment is presented.

### 2.5. RNA extraction, reverse transcription, and quantitative real-time PCR

Total RNA was extracted using Quick-RNA^TM^ MiniPrep Kit (Cat. No.: R1054. ZYMO Research, California, USA) according to manufacturer’s instructions. RNA concentration was determined by the absorbance at 260 nm using a Nanodrop One spectrophotometer (Thermo Fisher Scientific, Madison, USA). PCR reactions were performed on a BIOER PCR cycler machine using high-capacity cDNA reverse transcription kit (Applied Biosystems, USA). For quantitative mRNA expression, all experiments were performed in triplicates and an average of all three experiments is displayed. Gene expression was normalized to the housekeeping gene GAPDH using primers from Table 1 and analyzed through the using the Bio Rad CFX Manager software.

**Table 1:** Primers used for qRT-PCR.

Gene	F’ primer	R’ primer
*GAPDH*	GCGCACCGTCAAGGCTGAGAAC	AATGGTGGTGAAGACGCCAGT
*HSPA1A/B*	TTTGAGGGCATCGACTTCTACA	CCAGGACCAGGTCGTGAATC
*CLU*	TGCGGATGAAGGACCAGTGTGA	TTTCCTGGTCAACCTCTCAGCG
*CRYAB*	CCTGAGTCCCTTCTACCTTCG	CACATCTCCCAACACCTTAACTT
*ISG20*	ACACGTCCACTGACAGGCTGTT	ATCTTCCACCGAGCTGTGTCCA
*IFI27*	TGCTCTCACCTCATCAGCAGT	CACAACTCCTCCAATCACAACT
*IFIT2*	ATGGAGCAGATTCTGAGGCT	GAGGCTTCCAGACTCCAAAC
*IFI16*	TAGAAGTGCCAGCGTAACTCC	TGATTGTGGTCAGTCGTCCAT
*BATF2*	GCTGAAGAAGCAGAAGAACCGG	TGCAGGGACTGGATCTCCTTCC

### 2.6. Statistical analyses

Statistical analyses were performed using Microsoft Excel. Data are presented as mean ± SEM. Group means were compared using unpaired two-tailed Student’s t-tests. P values < 0.05 were considered statistically significant.

### 2.7. RNA-sequencing assays

#### 2.7.1. RNA extraction and QC.

Total RNA was extracted from two ovarian cancer cell lines, OVCAR4 and SKOV3. Three experiment repeats of each cell line were transfected with the plasmids GFP, Flag-AKT2 or Flag-BCAM-AKT2 (total of nine samples of each cell line). For RNA extraction the Qiacube (Qiagen) and RNeasy kits (cat no. 74106) were employed. Cells were disrupted in 350 ul buffer RLT and homogenized by needle and syringe. The lysate was then loaded on the Qiacube for automated extraction. The quality of the RNA was evaluated using the TapeStation 4200 (Agilent) with the RNA kit (cat no. 5067–5576). The RINe values of all samples were in the range of 9.7-10, indicating high quality.

#### 2.7.2. Library preparation and data generation.

Eighteen RNA-seq libraries were constructed simultaneously using NEBNext Ultra II Directional RNA Library Prep Kit for Illumina (NEB, cat no. E7760), according to the manufacturer’s protocol. Eight-hundred ng of total RNA was used as starting material. mRNA pull-down was performed using the NEBNext® Poly(A) mRNA Magnetic Isolation Module (NEB, cat no. E7490). RNA-seq library QC was performed by measuring library concentration using Qubit (Invitrogen) with the dsDNA HS Assay Kit (Invitrogen, cat no. Q32854) and size determination using the TapeStation 4200 with the High Sensitivity D1000 kit (cat no. 5067–5584). All libraries were then mixed into a single tube with equal molarity. The RNA-seq data was generated on Illumina NextSeq2000, using P2 100 cycles (Read1–100; Index1–8; Index2–8) (Illumina, cat no. 20046811).

#### 2.7.3. Bioinformatics analysis.

Quality control was assessed using Fastqc (v0.11.8). Reads were trimmed for adapters, minimum quality Phred score of 20 and a minimum length of 35 using CUTADAPT. Single-read FASTQs (100 bp) were aligned to the Human (GRCh38) reference genome and annotation file (http://ftp.ensembl.org/pub/release-91/gtf/homo_sapiens/Homo_sapiens.GRCh38.91.gtf.gz) using STAR with a mismatch ratio allowed < 0.2. The minimum and maximum intron sizes were set to 20 and 1,000,000, respectively. The number of reads per gene was counted using Htseq-count with ‘reverse’ mode. Normalization and differential expression analyses were conducted using DESeq2 R package. The similarity between samples was evaluated within DESeq2 package using an euclidean distance matrix (shown in a heatmap with a clustering dendrogram) and a principal component analysis (PCA). The latter is drawn from the most variable genes (50, 500, 1000, 2000, 5000, 10000 genes). An initial model of the data including all samples from both cell types presented an overwhelming amount of variance attributed completely to cell type. Therefore, it was decided to continue with two separate analyses for each cell type.

DESeq modeling: Since the experiment was carried out in separate batches (differing in dates performed and vials of thawed cells), the DESeq model was adjusted to batch. In the PCA analysis performed for samples of OVCAR4 cell line, one batch (#1) contributed substantial variance, which resulted in very few detected DEGs. Thus, to reduce the within-treatment group noise in the data, a model excluding batch #1 was applied in the DGE analysis. The threshold for significantly differentially expressed genes is determined by two factors: adjusted p-value ≤ 0.05 and the ‘base-mean independent filtering’ threshold, which is calculated by the DESeq2 algorithm for each comparison. FDR was calculated using the default approach of DESeq2, Benjamini-Hochberg.

## 3. Results

### 3.1 BCAM-AKT2 plays a proliferative role in HGSC

**Fig 1:**
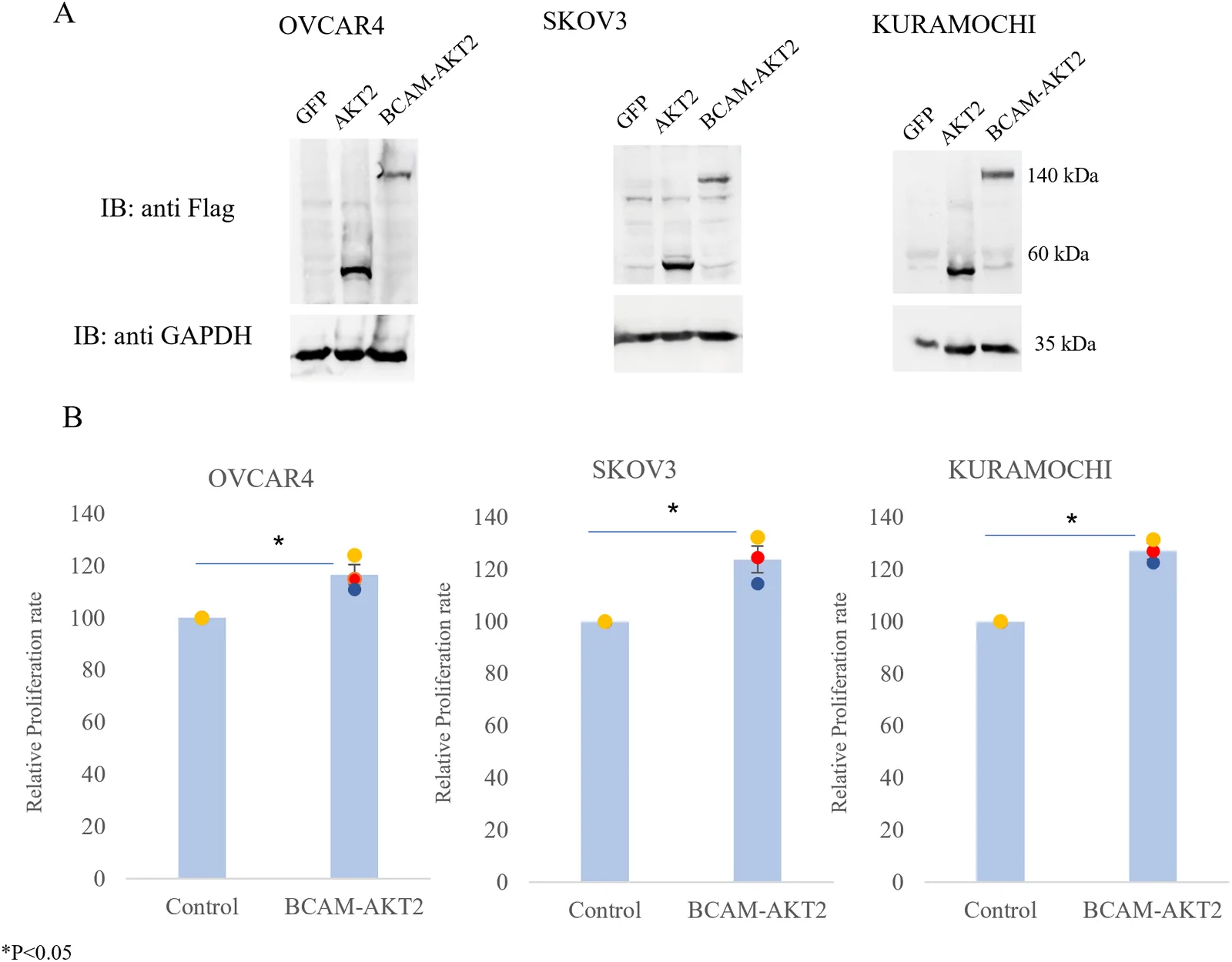
Effect of BCAM-AKT2 fusion protein on EOC cell lines. OVCAR4, SKOV3 and KURAMOCHI, cells were transiently transfected with BCAM-AKT2 fusion protein, AKT2 or GFP (empty vector). **(A)** Western blot analysis: cells were lysed and immunoblotted with the indicated antibodies following Western blot protocol. Results shown are representative of a typical experiment repeated three times. **(B)** Cell proliferation: XTT was conducted 3 days post-transfection in three experimental repeats. Relative proliferation rate is shown as fold change compared to GFP (empty vector) * p < 0.05.

Previous studies have shown that focus formation was highly correlated with a high level of BCAM-AKT2 expression [11]. To explore the proliferative role of BCAM-AKT2 in EOC, BCAM-AKT2 was overexpressed in KURAMOCHI, OVCAR4 and SKOV3 cells and proliferation was monitored by XTT assays. As seen in Fig 1, overexpression of BCAM-AKT2 led to increased proliferation in all three cell lines 72 hours post transfection compared to control (GFP).

### 3.2 BCAM-AKT2 effect on gene-expression in EOC cell lines

**Fig 2:**
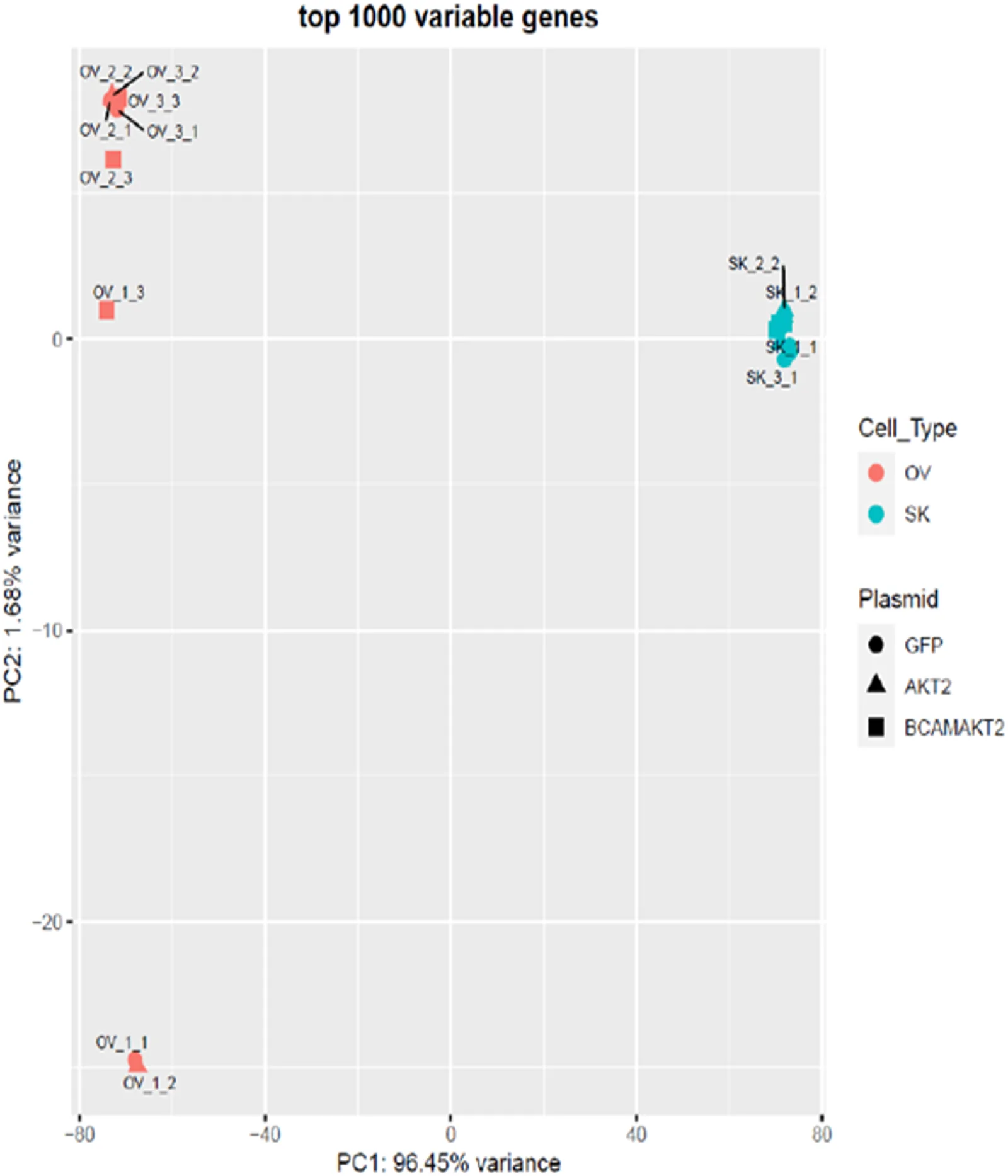
Principal component analysis (PCA) of OVCAR4 and SKOV3. Descriptive analysis of PCA plot of SKOV3 sample representing three experimental groups (Blue dot). Descriptive analysis of PCA plot of OVCAR4 samples representing three experimental groups (Pink dot).

To investigate the effect of BCAM-AKT2 on gene expression in EOC cell lines, RNA-seq was performed. To this end RNA was extracted from GFP-, Flag-AKT2- and Flag-BCAM-AKT2-transfected SKOV3 and OVCAR4 cells. In total, eighteen samples representing the two groups of ovarian cancer cell lines were transfected. Initially, RNA-sequencing data from all samples were analyzed using Principal Component Analysis (PCA) to assess overall similarities and differences among cell lines and treatment groups. As shown in Fig 2, samples clustered primarily according to cell line identity, with SKOV3 and OVCAR4 samples forming distinct and well-separated clusters. This separation indicates that the transcriptional differences between the two cell lines were a major source of variance in the dataset. Therefore, to avoid confounding effects arising from cell line-specific expression patterns and to better evaluate the transcriptional impact of BCAM-AKT2 within each cellular context, subsequent analyses were performed separately for each cell line.

**Fig 3:**
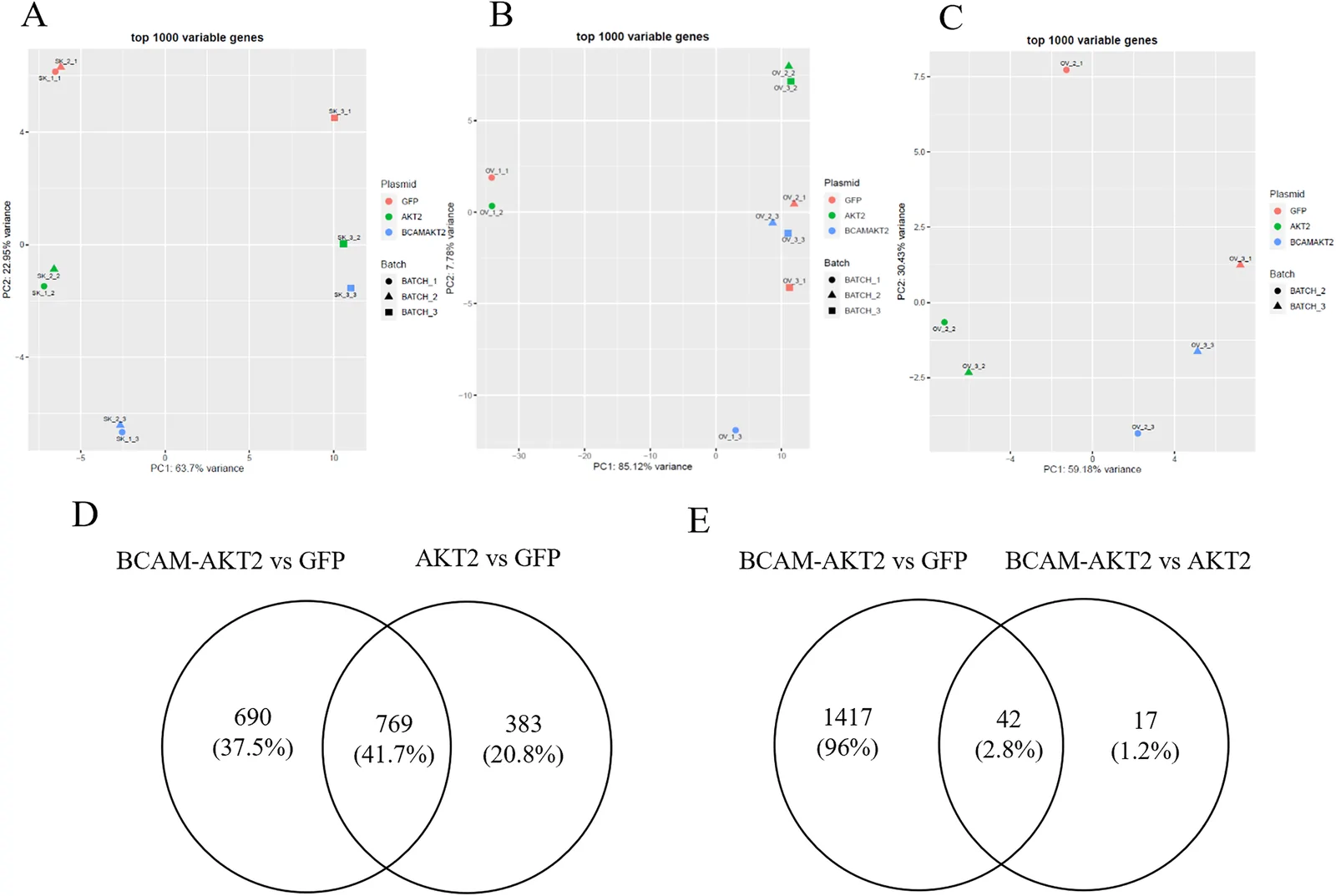
Exploring the effect of combined groups in SKOV3 and OVCAR4 through RNA-seq. (A) Descriptive analysis of PCA plot of SKOV3 sample representing three experimental groups. (B) Descriptive analysis of PCA plot of OVCAR4 samples representing three experimental groups, and (C) PCA of OVCAR4 after excluding one experiment. (D, E) Venn-diagram showing the intersections of differentially expressed genes in SKOV3.

According to the PCA data describing SKOV3 in Fig 3 there is good discrimination in the experiments repeats samples. Thus, PC1 (64%) presents differences between batches #1, #2 and #3, due to different experimental conditions. PC2 (23%) captures variability related to treatment (plasmid) (Fig 3A). The observed PC1 distance between the first two batches and the last one could be explained given different experimental conditions in SKOV3 with three experimental repeats.

**Fig 4:**
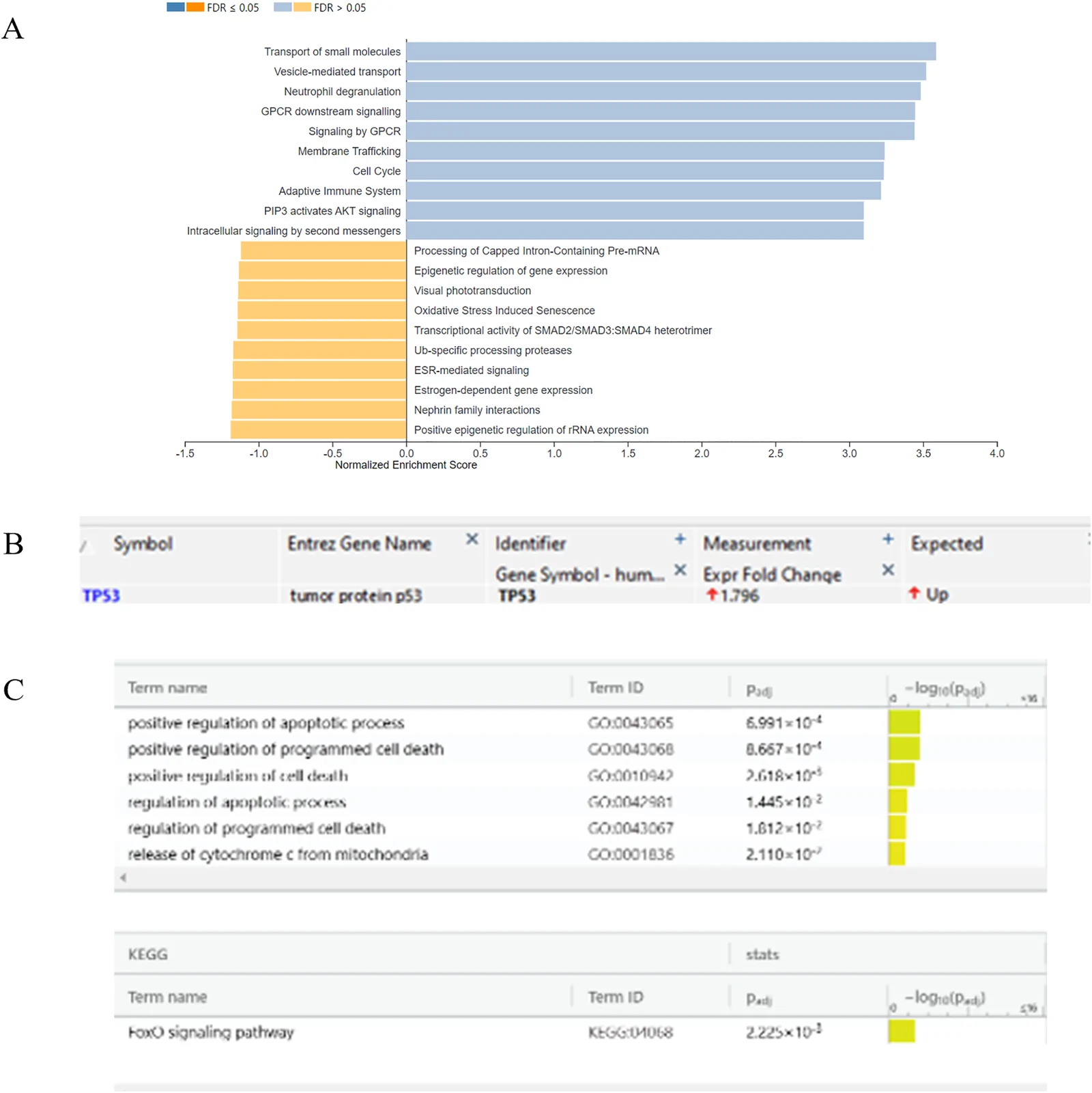
Gene Ontology of genes in SKOV3 (A) GSEA analysis of 769 common genes in SKOV3. (B) IPA analysis of 83 up-regulated genes out of 145 genes after applying a cutoff < −1.4 and >1.4-fold change to 769 genes. (C) GProfile of 62 down-regulated genes out of 145 genes after applying a cutoff < −1.4 and >1.4-fold change to 769 genes.

In OVCAR4, most of the variance in the data is represented by PC1 (85%), which distinguishes between experiment #1 and experiments #2,3 (again, related to thawing) (Fig 4B). When excluding experiment #1, treatment types (plasmid) are separable in PC1 x PC2 space (Fig 4C). The control plasmid is separated in both treatments in PC2 (30%). AKT2-transfected samples are separated from control and BCAM-AKT2 on PC1 (60%). Based on the results of these initial analyses, we chose to focus our analysis on the OVCAR4 cell line only with batches #2,3 and to exclude batch #1.

As alluded to above, OVCAR4 and SKOV3 are two models of ovarian cancer cell lines that differ in their genomic profiles. There is a significant difference in the molecular profiles between the two in vitro tumor models, as SKOV3 represents a “less likely” high-grade serous cell line whereas OVCAR4 represents a more “likely” high-grade serous cell line [15].

Next, we examined the extent of the difference in gene expression between the different treatment groups. In agreement with the cell lines phenotypic features, the effect of either BCAM-AKT2 or AKT2 on total gene expression was more profound in SKOV3 than in OVCAR4. In SKOV3 cells, 9%, 11.5% and 0.35% of the total tested genes were differentially expressed (DEG`s) in AKT2 vs. GFP, BCAM-AKT2 vs. GFP and BCAM-AKT2 vs. AKT2, respectively. In OVCAR4 cells only 1.6%, 0.03% and 1.13% of the total tested genes were differentially expressed in AKT2 vs. GFP, BCAM-AKT2 vs. GFP and BCAM-AKT2 vs. AKT2 respectively.

Additionally, we discriminated between up- or down-regulated DEGs. Consequently, in SKOV3 both BCAM-AKT2 and AKT2 vs GFP transfections resulted in up-regulation of 66% of DEGs and down-regulation of 34% of DEGs. In OVCAR4 we found that AKT2 vs GFP treatment induced mainly DEGs which were up regulated (91%). Interestingly, BCAM-AKT2 had no effect on gene expression in the OVCAR4 model.

Next, we were interested to explore the effect of BCAM-AKT2 compared to that of AKT2 in SKOV3. For this purpose, we intersected by Venn diagram all 1152 DEGs affected by AKT2 relative to GFP with 1459 DEGs affected by BCAM-AKT2 relative to GFP. Notably, among the non-overlapping DEGs, 690 genes differed only in respect to BCAM-AKT2 and 769 common DEG`s to AKT2 and BCAM-AKT2 (Fig 4D). In addition, by applying a cutoff of either below or above 1.4-fold change, 769 DEGs and 690 DEGs were filtered to 145 DEGs and 121 DEGs, respectively. Out of the 145 genes, 83 were upregulated and 62 downregulated genes. Furthermore, intersection of the 1459 DEGs of BCAM-AKT2 relative to GFP with 59 DEGs of BCAM-AKT2 relative to AKT2 yielded 42 genes that were regulated uniquely by BCAM-AKT2.

**Fig 5:**
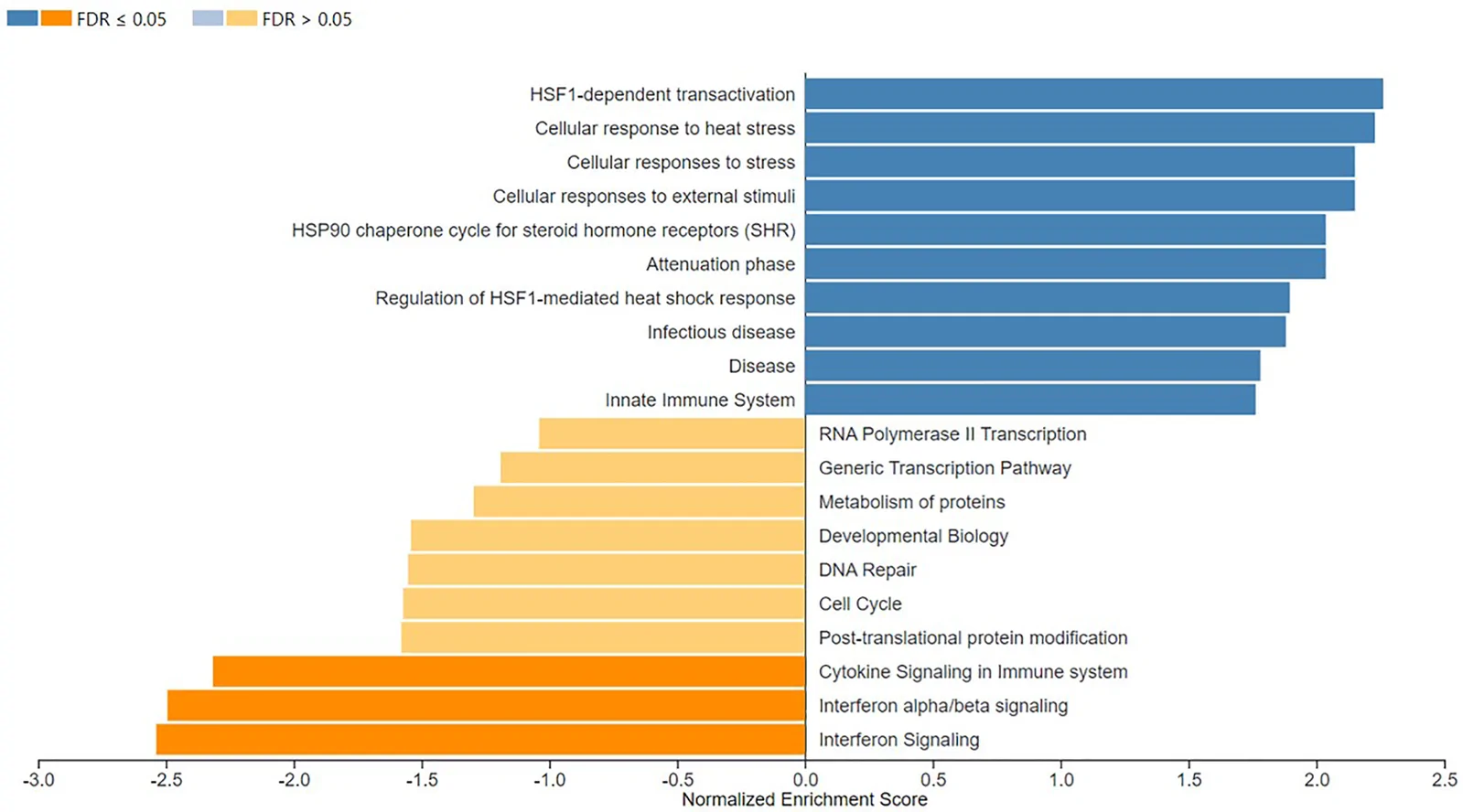
Gene Ontology of genes in SKOV3. GSEA analysis of 121 genes after applying a cutoff < −1.4 and >1.4-fold change to 690. genes.

Next, we performed Gene Ontology (GO) analysis on the above genes to identify enriched biological processes. For this purpose, we used Ingenuity Pathway Analysis (IPA) and Gene Set Enrichment Analysis (GSEA). Applying GSEA (Webgestalt on-line tool) to the 769 DEGs showed no significant pathways enrichment. In addition, looking specifically on IPA analysis on the predicted top transcriptional regulators that are activated by the mutual upregulated genes in SKOV3 we identified the TP53 gene (fold change = 1.79. p = 4.30E17, Zcore = 3.1) in both BCAM-AKT2 and AKT2 groups (Fig 4B). On the other hand, the down regulated genes were mainly involved in the FOXO signaling pathway (Zscore = 2.449, pvalue = 1.11E-E02), and in positive regulation of apoptosis (G: Profiler) (Fig 5C).

Next, we examined the 690 DEGs and 121 DEGs that were derived following a cutoff of either below or above 1.4-fold change. Interestingly, running 690 DEGs which were unique to BCAM-AKT2 by IPA, revealed enrichment of canonical pathways that were either activated or inhibited. Specifically, among the top inhibited pathways were Interferon Signaling, Antiviral Response and Pyropstosis Signaling Pathways, while Unfolded Protein Response was the most enriched activated pathway (Table 2).

**Table 2:** IPA analysis of canonical pathways of 690 genes unique to BCAM-AKT2 in SKOV3.

Inhibited	Activated
Antiviral Response (p_v_ =4.48E×10^-2^, Z_Score_= -2.530)	Unfolded Protein Response (p_v_ = 3.68E×10^-5^, Z_Score_= 2.449)
Interferon Signaling (p_v_ =2.9E×10^-3^, Z_Score_= -2.236)	
Pyroptosis Signaling Pathway (p_v_ =1.44E×10^-2^, Z_Score_= -1.890)	

Furthermore, through GSEA analysis of 121 DEGs, Interferon Signaling was detected among the most inhibited pathways (Fig 5).

**Fig 6:**
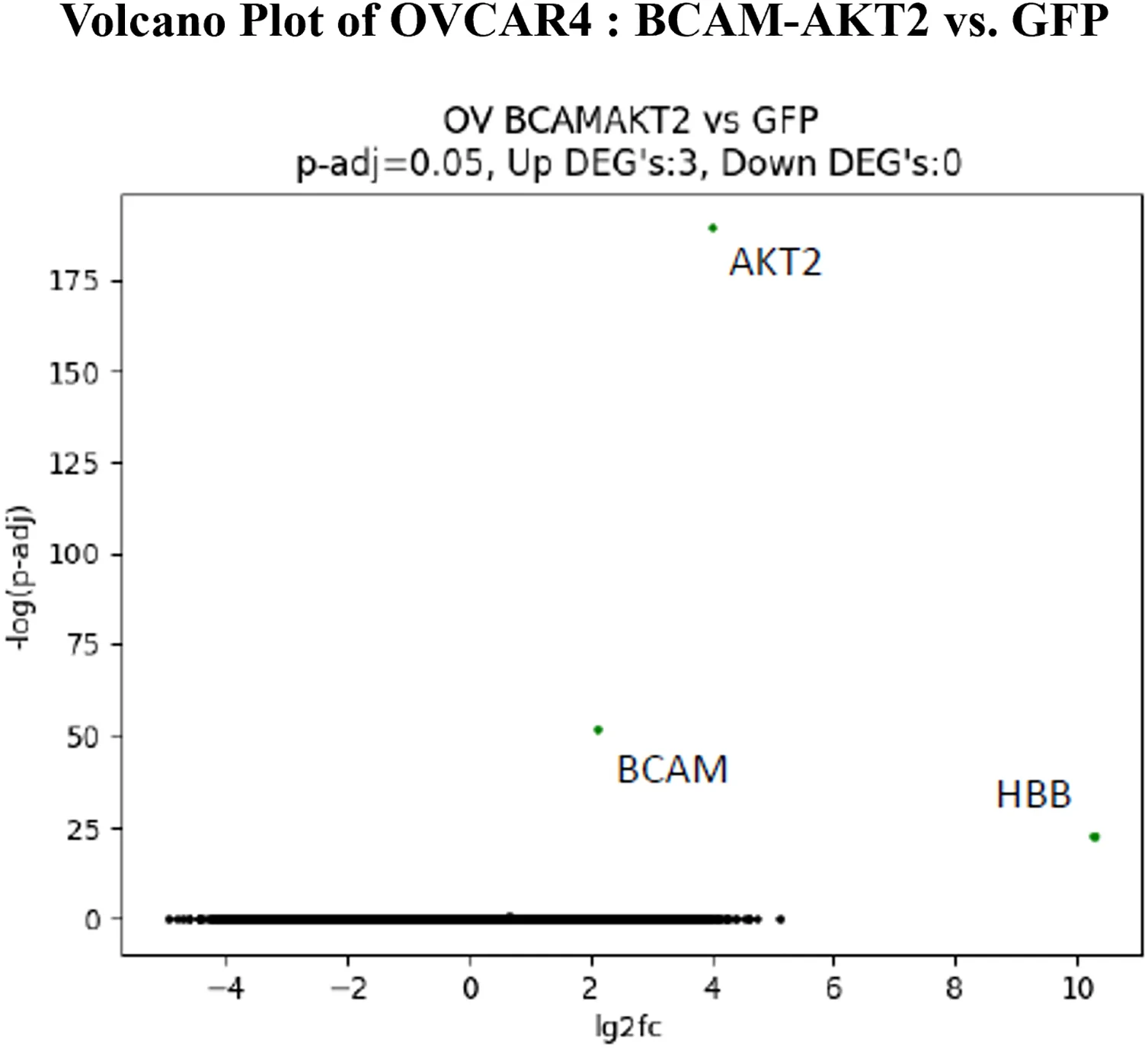
Volcano Plot of BCAM-AKT vs GFP in OVCAR4. The x-axis shows the fold-change in gene expression between two groups (BCAM-AKT2 vs. GFP) in OVCAR4, y-axis shows the statistical significance of the differences. Each dot represents a gene: dots marked in black represent genes without significant differential expression.

As compared to the SKOV3 cell line, OVCAR4 exhibited high levels of endogenous *BCAM* gene expression. As a result of this enhanced expression, exogenous BCAM-AKT2 had no major effect on the gene expression in OVCAR4 relative to GFP. Looking at OVCAR4, it is important to note that BCAM-AKT2 vs GFP comparison resulted in only 3 upregulated genes: *AKT2, BCAM* and *HBB* as seen in the Volcano plot (Fig 6 and Table 3).

**Table 3:** Summary of pair-wise differential expression analysis – All samples dataset.

Dataset	Comparison name	Total Genes	All Zero	Low Counts	Tested	Significant Up	Significant Down	Total DE Genes
	AKT2 vs. GFP	58243	28658	16874	12711	755	397	1152
	BCAM-AKT2 vs. GFP	58243	28658	16874	12711	957	502	1459
	BCAM-AKT2 vs. AKT2	58243	28658	12780	16805	22	37	59
	AKT2 vs. GFP	58243	30788	14024	13431	198	20	218
	BCAM-AKT2 vs. GFP	58243	30788	14024	10604	3	0	3
	BCAM-AKT2 vs. AKT2	58243	30788	14024	16028	39	142	181

**Fig 7:**
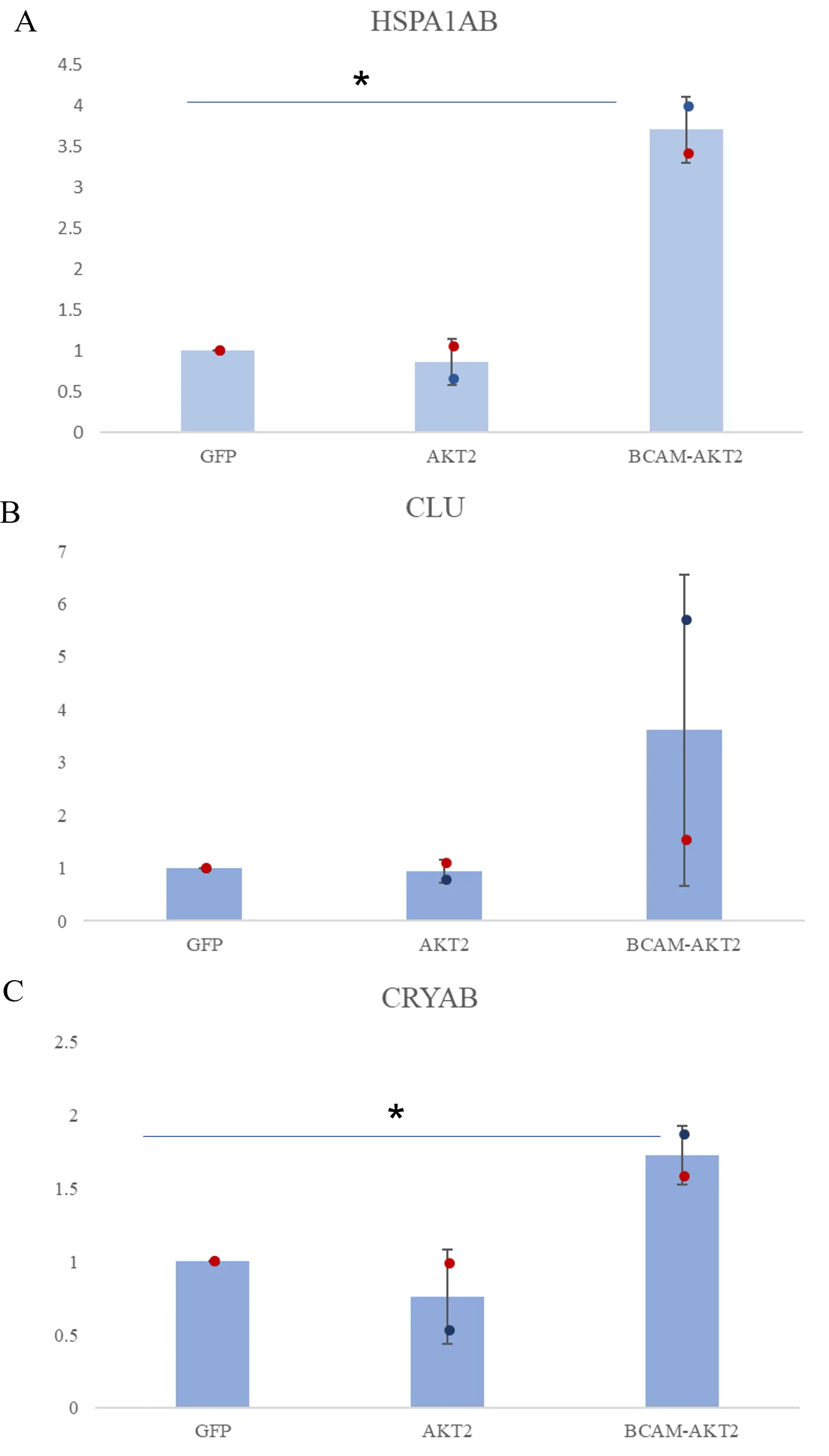
qRT-PCR validation experiments in SKOV3. SKOV3 cells were transfected with the plasmids BCAM-AKT2 fusion protein, AKT2 or GFP (empty vector) for 48 hours. Then, quantitative real-time PCR was conducted for validation experiments. Genes (A + C) were increased compare to GFP control and in correlation with RNA-seq results Data are presented as mean ± SEM. Individual data points representing biological replicates are overlaid on the bar plots and each dot represents an individual biological replicate. Statistical significance was determined using unpaired two-tailed Student's t-tests comparing. *P < 0.05.

To validate the RNA sequencing data, we assessed the expression of genes associated with two distinct signaling pathways: interferon signaling, which was downregulated, and the unfolded protein response (UPR), which was upregulated. SKOV3 cells were transfected with GFP, Flag-AKT2, or Flag-BCAM-AKT2 plasmids for 48 hours, followed by quantitative real-time PCR (qRT-PCR) using gene-specific primers (Table 1). qRT-PCR analysis confirmed the RNA-seq results. As shown in Fig 7, UPR-related genes including *CRYAB* and *HSPA1AB* were significantly upregulated in cells transfected with Flag-BCAM-AKT2 compared to the GFP control.

**Fig 8:**
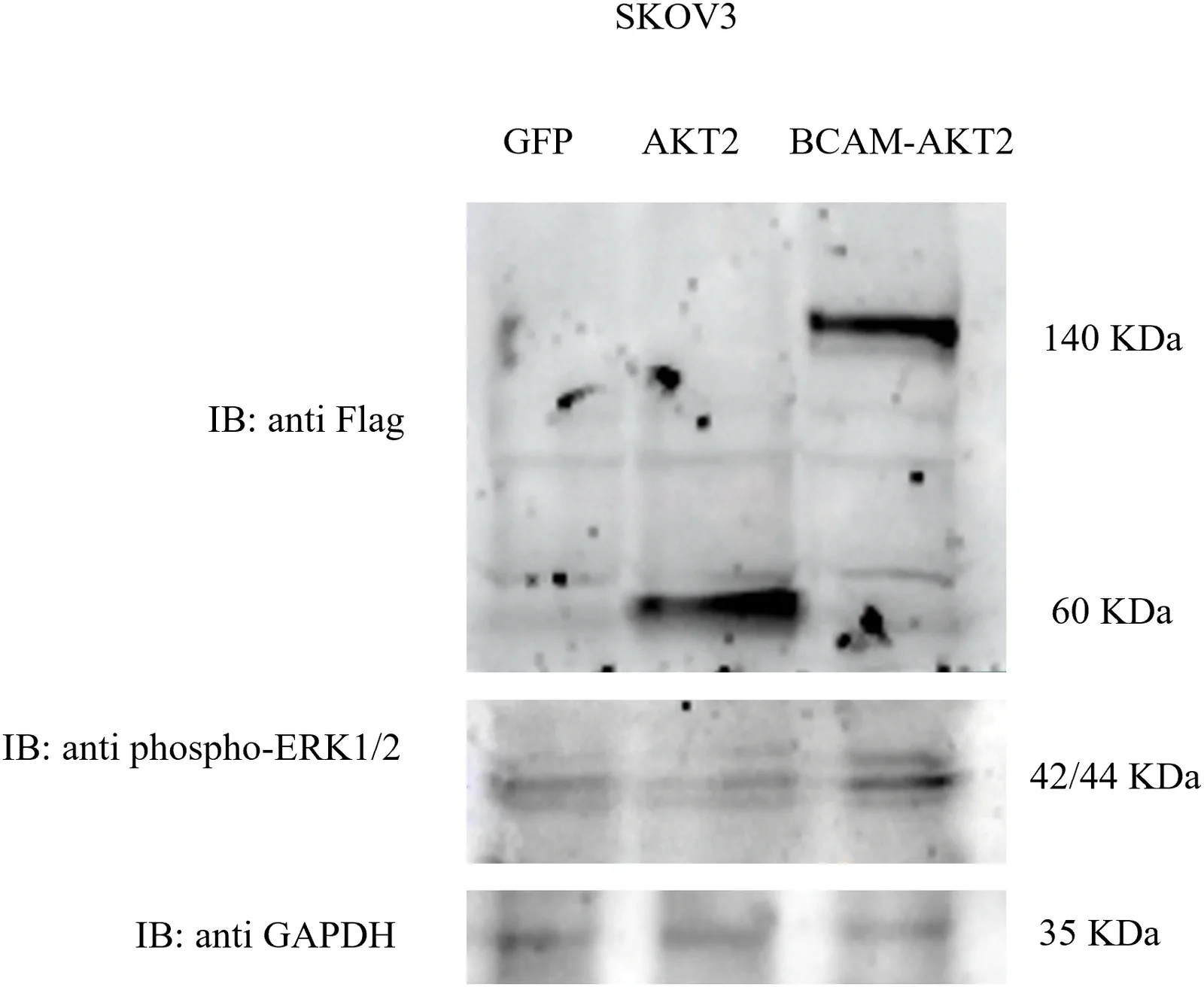
Effect of BCAM-AKT2 fusion protein on phospho-ERK1/2 in SKOV3 cells. Cells were transiently transfected with BCAM-AKT2 fusion protein, AKT2 or GFP (empty vector). Cells were lysed and immunoblotted with the indicated antibodies following Western blot protocol. Results shown are representative.

To further evaluate signaling pathway modulation following BCAM-AKT2 expression, Western blot analysis of phospho-ERK1/2 was performed in SKOV3 cells after transfection. A modest increase in phospho-ERK1/2 levels was observed in BCAM-AKT2-transfected cells compared with control and AKT2 transfected cells. Notably, basal phospho-ERK1/2 expression was detected in both control and AKT2-transfected cells, indicating constitutive ERK activity in SKOV3 cells (Fig 8).

## 4. Discussion

It is estimated that 70% of all women diagnosed with ovarian cancer will die from the disease [16]. In most cases, patients initially respond to surgery and/or chemotherapy, but the disease eventually relapses and at that point there is a lack of good therapeutic options. The PI3K-AKT signaling pathway controls a variety of essential cellular functions, including cell proliferation, survival, metabolism, motility, and responses to stress. It is the second most frequently altered pathway in EOC [17, 18]. Given its association with chemotherapy resistance and poor prognosis, this pathway is sought as a potentially useful therapeutic target for EOC [19].

There are several types of cancers for which fusion proteins are used for diagnosis [20]. One specific fusion protein in prostate cancer is TMPRSS2: ERG, a transmembrane protease fusion transcript that has been identified as a promising urinary biomarker [21]. In a similar manner, the BCR-ABL fusion protein is associated with hematological malignancies and serves as a model of successfully overcoming resistance to cancer therapy [22]. BCR-ABL serves as a paradigm for the targeting of oncogenic tyrosine kinase signaling pathways in leukemia [23]. In addition, Banerji et al. had identified MAGI3-AKT3 as a novel oncogene in breast cancer, and that its oncogenic activity is caused by disruption of the PI3K/AKT pathway. The MAGI3–AKT3 fusion leads to constitutive activation of AKT kinase in breast cancer [24].

In view of the rapidly increasing evidence that gene fusion mutations are frequent drivers of cancer, a deeper understanding of the molecular and functional hallmarks of specific gene fusions is essential. In this work we aimed to identify the molecular signature of the BCAM-AKT2 fusion protein in ovarian cancer cell lines. The fact that BCAM-AKT2 is constitutively phosphorylated may suggest that its expression is regulated by the BCAM promoter as opposed to endogenous AKT2, since phosphorylation of BCAM-AKT2 is no longer dependent on external stimuli.

Several carcinogenic processes are facilitated by the interaction between cancer cells and extracellular matrix (ECM), including cell proliferation, survival, migration, homing, and metastasis [25]. This has led to the notion that adhesion molecules are potential therapeutic targets. BCAM is a glycoprotein that belongs to the immunoglobulin superfamily and contains both the Lu blood group and BCAM tumor-associated antigens [26]. There are two isoforms of the transmembrane BCAM protein: (628 amino acids) and (v13) (588 amino acids), which differ in size. BCAM contains binding sites for SH3 domains and phosphorylation for signal transduction. Increasing evidence suggests that BCAM contributes to tumorigenesis [27]. Specifically, many types of cancer have been shown to exhibit BCAM expression, including colon, skin, brain, liver, thyroid, breast, and bladder cancers [26, 28]. Guadall et al. reported that BCAM promotes tumor cell migration by binding to laminin α5 chain on epithelial cells [29]. Also, Määttä et al. reported that non-polarized expression of BCAM promotes local tumor progression in epithelial ovarian cancer tissues [30]. To date, the underlying molecular mechanisms and downstream signaling pathways of BCAM in tumorigenesis are still unknown.

Many signal transduction pathways are constitutively activated in cancer, with ensuing cell proliferation [31]. Using XTT proliferation assays, we showed that overexpressed BCAM-AKT2 induces proliferation in all three cell lines: SKOV3, KURAMOCHI and OVCAR4 (Fig 1). In KURAMOCHI there was a 27% increase in proliferation of BCAM-AKT2-transfected cells compared to GFP, while in SKOV3 there was a 24% increase and in OVCAR4 18%, referring to three experimental repeats in each cell line.

Next, through RNA-seq analysis of OVCAR4 and SKOV3 cell lines, we aimed to investigate the effect of BCAM-AKT2 on gene expression. According to the genomic data analysis published by Domcke et al. the OVCAR4 and KURAMOCHI cell lines are regarded as representative of “high-grade serous ovarian cancer” (HGSOC) whereas SKOV3 belongs to a category of “unlikely high-grade serous carcinoma” [15]. HGSOC is the most common and aggressive form of epithelial ovarian cancer, demonstrating a markedly higher metastatic potential and rapid clinical progression compared to other type of epithelial ovarian cancer [32]. The RNA-seq analysis showed that OVCAR4 cells have high expression level of endogenous BCAM, as opposed to SKOV3 cells that showed low level of endogenous expression. This interesting finding is in line with recent growing evidence that BCAM plays a role in cell adhesion and migration, as well as tumor metastasis. BCAM may be associated with different types of cancer including epithelial skin cancer, ovarian cancer, bladder cancer, and gastric cancer [26]. BCAM mediates cell adhesion and migration and has been reported to play a functional role in thyroid and gastric cancers metastases [33]. Schon et al. demonstrated that 27 of 31 epithelial skin tumors were BCAM-positive and provided evidence that the protein was distributed uniformly and with a non-polarized pattern [28].

Beyond basal BCAM expression, the limited number of DEGs observed in OVCAR4 cells may reflect saturation of BCAM-associated signaling pathways, cell line-specific signaling context, or compensatory mechanisms that attenuate downstream transcriptional responses. In this setting, exogenous expression of BCAM-AKT2 may represent a relatively mild perturbation, in contrast to SKOV3 cells, where BCAM-AKT2 introduction induces a robust transcriptomic response.

RNA-seq analysis suggested that BCAM-AKT2 expression in SKOV3 cells is associated with activation of the UPR and potential inhibition of interferon alpha/beta signaling pathways. qRT-PCR validation confirmed a significant upregulation of UPR-related genes, supporting the involvement of this pathway.

However, the anticipated downregulation of *interferon signaling* genes was not validated at the mRNA level. One possible explanation is the inherently low basal expression of many interferon-stimulated genes in these cells, which can hinder the reliable detection of further downregulation using qRT-PCR. This technical limitation may reduce sensitivity and mask subtle expression changes. Additionally, discrepancies between RNA-seq and qRT-PCR data can arise due to differences in dynamic range, normalization methods, and primer specificity.

The UPR is a cellular adaptive mechanism activated by the accumulation of unfolded or misfolded proteins in the endoplasmic reticulum (ER). This accumulation can result from stress conditions such as oxidative stress, nutrient deprivation, calcium imbalance, or viral infection. The UPR works to restore ER homeostasis by temporarily reducing protein synthesis, enhancing the folding capacity of the ER, and promoting the degradation of misfolded proteins. However, under prolonged or severe stress, UPR signaling can trigger apoptosis [34]. Notably, sustained activation of the AKT2-PI3K axis is known to increase protein synthesis and proteostatic load, which can induce ER stress and activate the UPR [35].

In cancer, UPR signaling is frequently adopted as a survival mechanism to support tumor cell proliferation and angiogenesis [36]. Also, activation of UPR can result in reduced sensitivity to chemotherapy and resistance to treatment as demonstrated by Salaroglio et al. [37]. In addition, Yart et al. identified a link between the UPR system and ovarian cancer. Specifically, they showed that UPR activation increased the in vitro formation of polyploid giant cancer cells by cell fusion in ovarian cell lines [38].

Overall, our RNA seq results indicated that BCAM-AKT2 modulated UPR signaling pathway, which is known to interact with oncogene and tumor suppressor networks during cancer development.

To further explore signaling pathways associated with the UPR signature identified by RNA-seq, we assessed phospho-ERK1/2 expression following BCAM-AKT2 transfection. A slight increase in phospho-ERK1/2 protein levels was detected following BCAM-AKT2 over expression. Basal ERK1/2 phosphorylation was also observed in control and AKT2-transfected cells, indicating that ERK signaling is constitutively active in SKOV3 cells. This basal activation may account for the relatively moderate increase detected following BCAM-AKT2 transfection. Importantly, ERK signaling has been previously linked to activation of the UPR pathway [36]. Therefore, the observed elevation in phospho-ERK1/2 levels may further support our RNA-seq findings indicating activation of UPR-related pathways in BCAM-AKT2-expressing cells.

***In conclusion***, our study suggests that the BCAM-AKT2 fusion protein is involved in the regulation of ovarian cancer cell proliferation and gene expression. The constitutive activation of BCAM-AKT2, reflected by sustained phosphorylation, points to a disturbance in normal cellular regulatory mechanisms and indicates that this fusion protein may contribute to oncogenic signaling in epithelial ovarian cancer. The observed increase in cell proliferation supports a role for BCAM-AKT2 in promoting tumor cell growth.

Gene expression analyses revealed alterations in several pathways, including activation of the UPR-related genes, which were observed specifically in SKOV3 cells. These findings suggest that BCAM-AKT2 may influence cellular stress responses and tumor cell adaptation, potentially contributing to tumor survival. Our results emphasize the importance of further investigating the molecular mechanisms associated with BCAM-AKT2 and its downstream signaling pathways. Future studies are needed in order to validate these findings using additional *in vivo* models and clinical samples, as well as to evaluate the therapeutic potential of targeting the BCAM–AKT2 axis in epithelial ovarian cancer.

## Supporting information

S1 Raw images Original western blot images. (PDF)

S1 Dataset Raw RNA sequencing data underlying the findings of this study. (CSV)
