# Supplementary material for: BCAM-AKT2 fusion protein promotes unfolded protein response driven oncogenic signaling in SKOV3 epithelial ovarian cancer cell line
Source: PLoS One. 2026 Sep 25;21(9):e0357882. doi: 10.1371/journal.pone.0357882 (PMC13614538; doi:10.1371/journal.pone.0357882)
Supplement: Supplementary file 1 [file pone.0357882.s001.pdf]

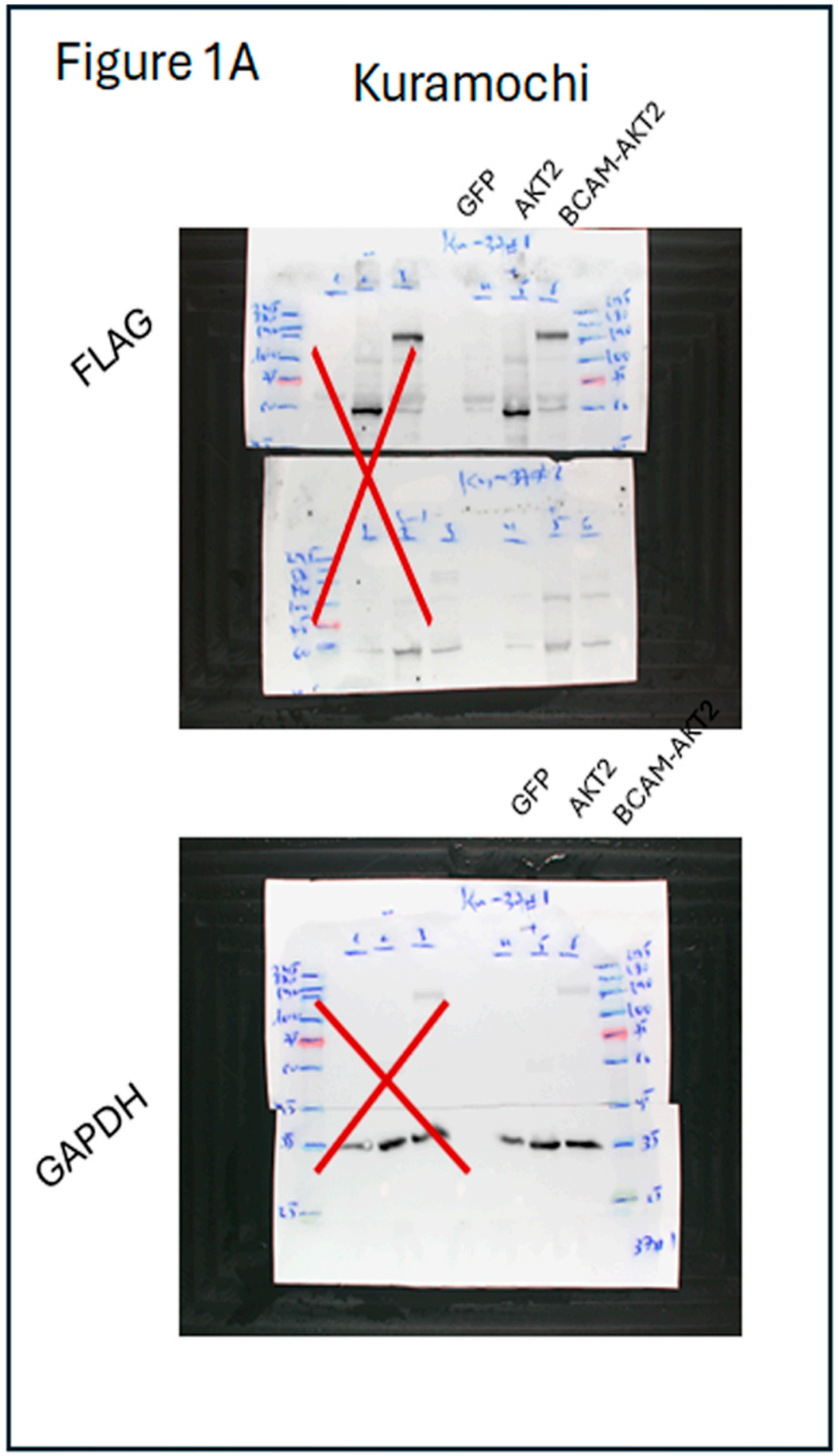
Figure 1A
Kuramochi
GFP
AKT2
BCAM-AKT2
FLAG
GAPDH

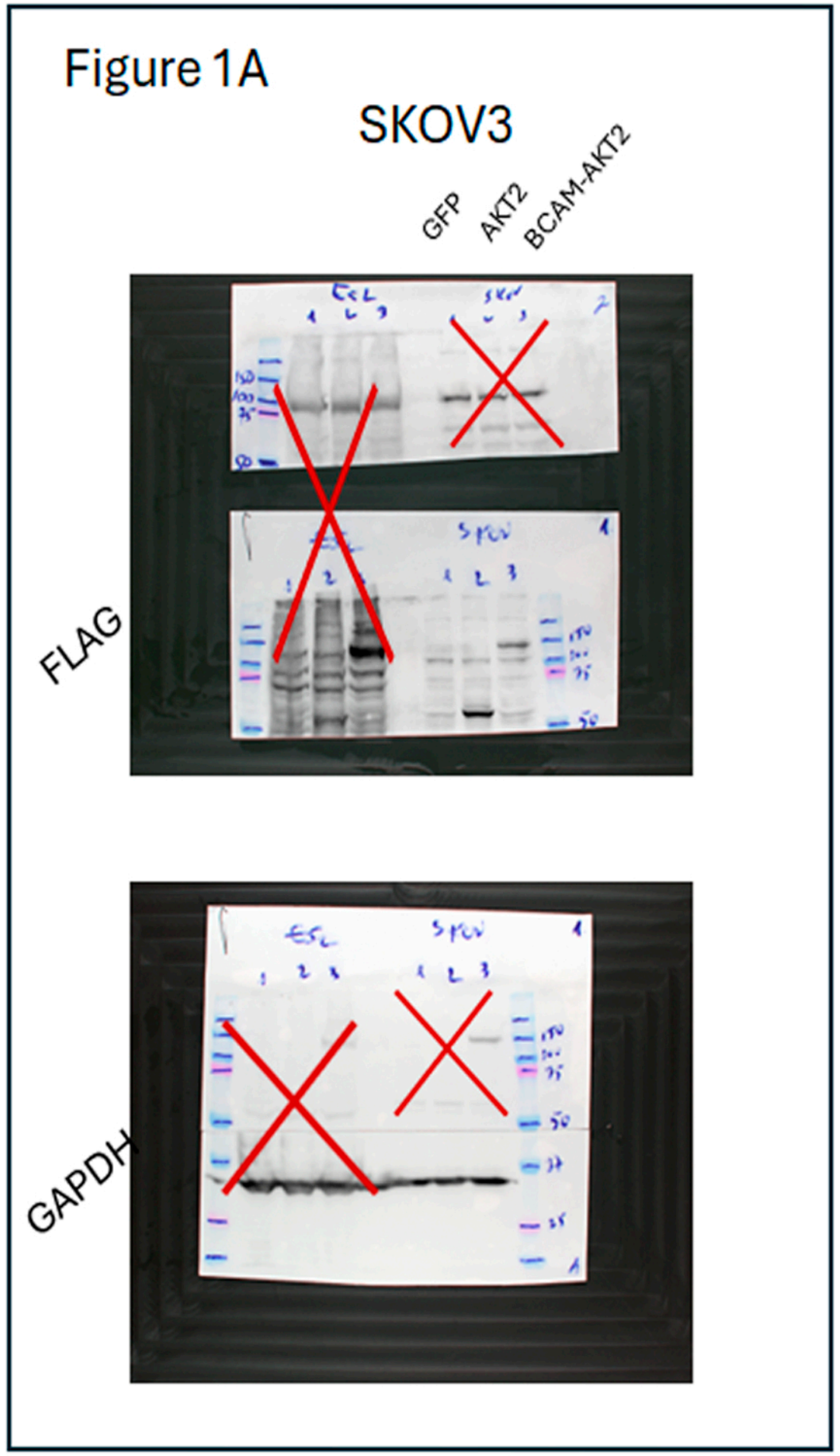
Figure 1A
SKOV3
GFP
AKT2
BCAM-AKT2
FLAG
GAPDH

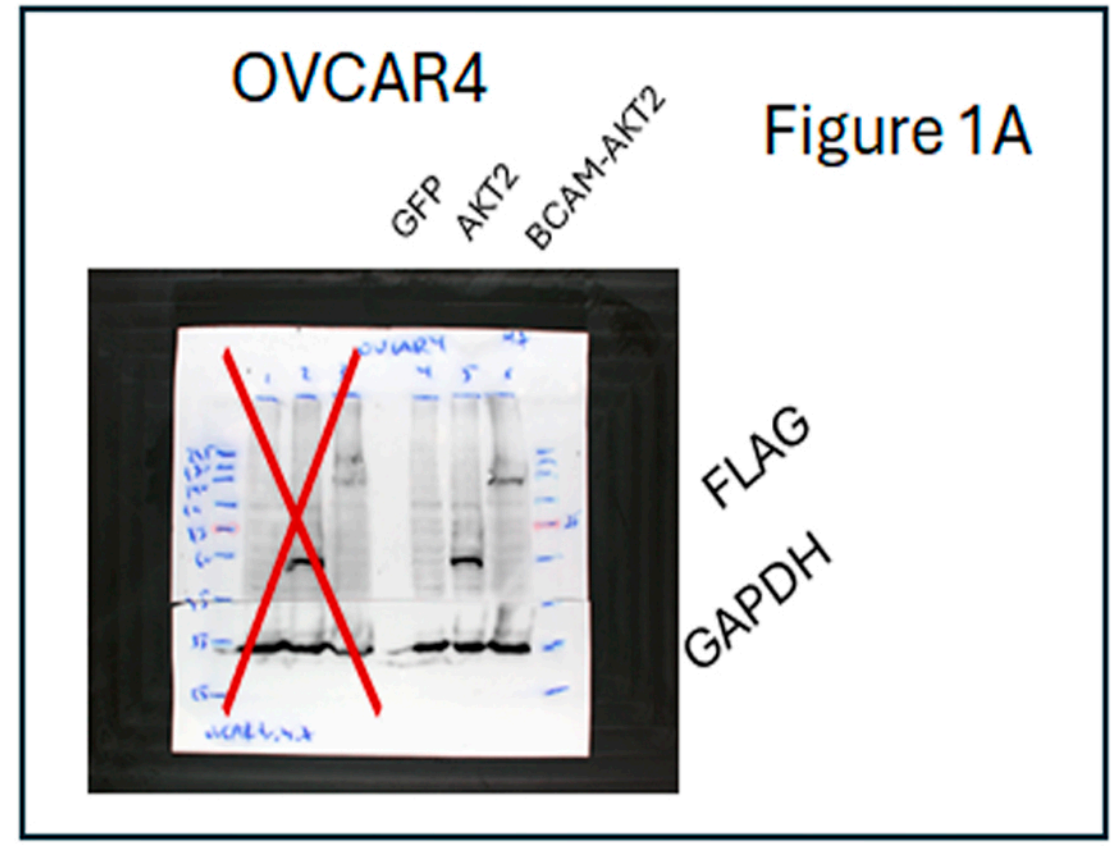
OVCAR4
Figure 1A
GFP
AKT2
BCAM-AKT2
FLAG
GAPDH

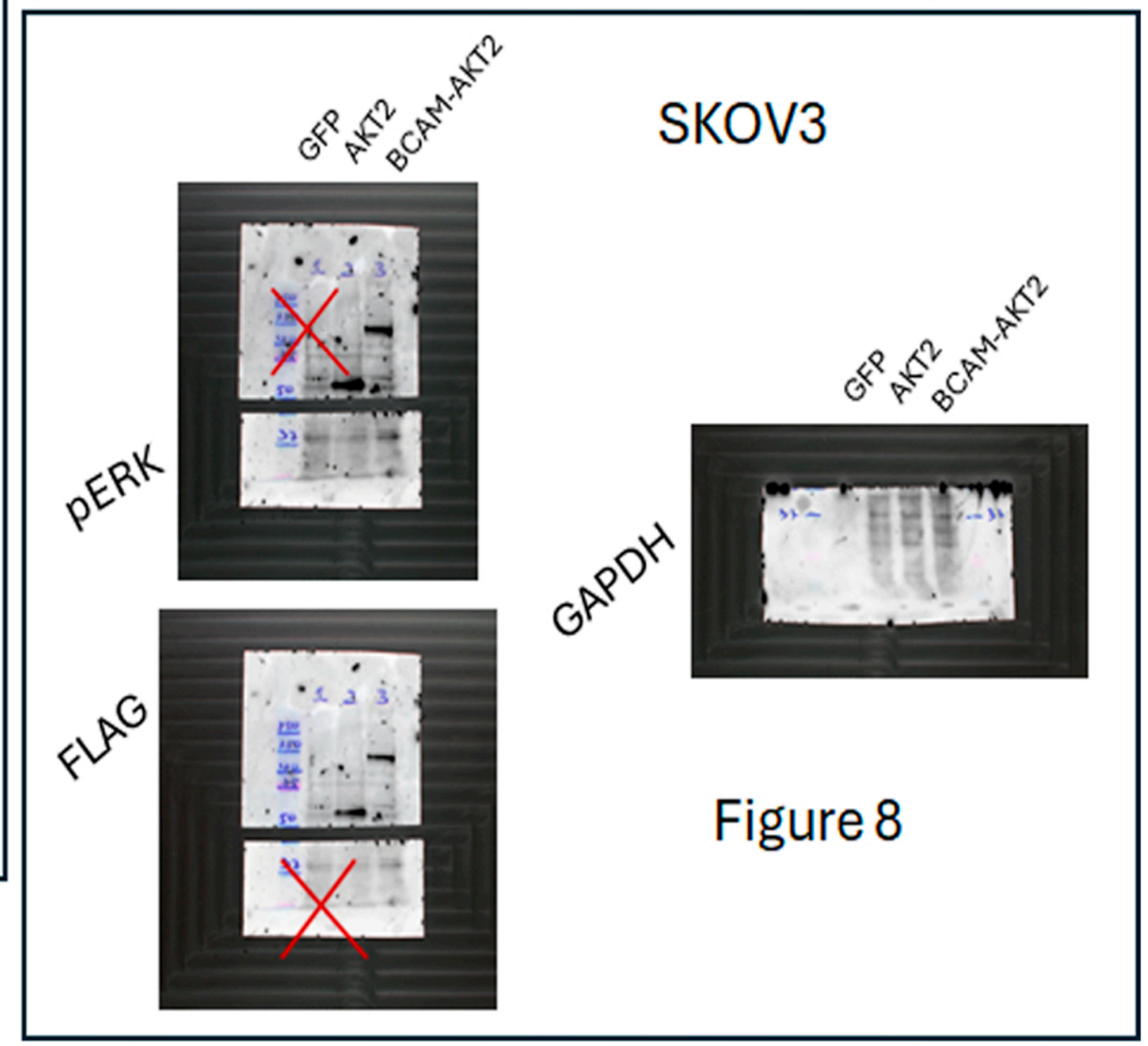
SKOV3
GFP
AKT2
BCAM-AKT2
pERK
FLAG
GAPDH
Figure 8
